# Outcomes of inpatient chemoradiotherapy for patients with newly diagnosed glioblastoma and significant functional impairment

**DOI:** 10.1093/nop/npaf109

**Published:** 2025-10-24

**Authors:** Neil Sielski, Anne S Reiner, William E Rosa, Brandon S Imber, Luke R G Pike, Louis P Voigt, Lauren R Schaff, Andrew L Lin, Christian Grommes, Igor T Gavrilovic, Jacqueline Stone, Alan C Carver, Marc Rosenblum, Viviane Tabar, Thomas Kaley, Ingo K Mellinghoff, Elena Pentsova

**Affiliations:** Department of Neurology, Memorial Sloan Kettering Cancer Center, New York; Department of Epidemiology and Biostatistics, Memorial Sloan Kettering Cancer Center, New York; Department of Psychiatry and Behavioral Sciences, Memorial Sloan Kettering Cancer Center, New York; Department of Radiation Oncology, Memorial Sloan Kettering Cancer Center, New York; Department of Radiation Oncology, Memorial Sloan Kettering Cancer Center, New York; Department of Anesthesiology, Pain & Critical Care Medicine, Memorial Sloan Kettering Cancer Center, New York; Department of Neurology, Memorial Sloan Kettering Cancer Center, New York; Department of Neurology, Memorial Sloan Kettering Cancer Center, New York; Department of Neurology, Memorial Sloan Kettering Cancer Center, New York; Department of Neurology, Memorial Sloan Kettering Cancer Center, New York; Department of Neurology, Memorial Sloan Kettering Cancer Center, New York; Department of Neurology, Memorial Sloan Kettering Cancer Center, New York; Department of Pathology and Laboratory Medicine, Memorial Sloan Kettering Cancer Center, New York; Department of Neurosurgery, Memorial Sloan Kettering Cancer Center, New York; Department of Neurology, Memorial Sloan Kettering Cancer Center, New York; Department of Neurology, Memorial Sloan Kettering Cancer Center, New York; Department of Neurology, Memorial Sloan Kettering Cancer Center, New York

**Keywords:** clinical decision-making, functional disability, glioblastoma, palliative care, patient-centered outcomes

## Abstract

**Background:**

Clinical outcomes in patients with glioblastoma and low Karnofsky Performance Scale (KPS) in the inpatient setting are rarely described. Limited survival data and high symptom burden complicate decision-making. This study describes patient-centered outcomes in functionally debilitated patients with newly diagnosed glioblastoma following cancer-directed treatment.

**Methods:**

We retrospectively reviewed charts of patients with newly diagnosed glioblastoma who received radiotherapy (RT) while hospitalized. Patient, tumor, and treatment characteristics were included in univariable Cox regression and logistic models to assess associations with overall survival (OS) and discharge home. Multivariable models included factors significant on univariable analysis. OS was estimated by the Kaplan-Meier method.

**Results:**

Of 52 patients (median age: 68 years, range: 21-85), median KPS was 50 (range: 30-60). All underwent RT and 50 (96%) received concurrent temozolomide; 12 (23%) received bevacizumab, and 46 (88%) completed planned RT. Median length of stay (LOS) was 29 days (range: 20-65). Only 40% of patients were able to participate in decision-making. Specialty palliative care consultation was provided to 16 (31%). Median OS was 5.82 months. In multivariable analysis, lower KPS remained the sole factor significantly associated with increased risk of death (*P* = .002) and decreased odd of discharge home (*P* = .02).

**Conclusion:**

Inpatient treatment for functionally disabled patients with glioblastoma was associated with prolonged LOS and poor outcomes. These findings highlight opportunities for early incorporation of specialty palliative care to multimodal cancer-directed therapies to optimize symptom management, elicit patient values and preferences, and facilitate goal-concordant decision-making. Prospective studies are necessary to identify predictors of functional recovery and treatment options.

Key Points

- Patients with newly diagnosed glioblastoma and low KPS display poor survival and prolonged length of stay.
- Quality of life, patient and caregiver experience, and decision-making provide insights beyond survival.

Importance of the Study

This retrospective review describes patient-centered outcomes in functionally debilitated patients with newly diagnosed glioblastoma following cancer-directed treatment. We hypothesize that abbreviated cancer-directed treatment could improve overall quality of life (QOL) without significant impact on overall survival and offer an opportunity for early palliative care intervention. Our results suggest that patients with low Karnofsky Performance Status could benefit from QOL-centered studies, where survival outcomes might not be amenable to improvement with experimental treatments, but where length of stay could be substantially decreased. Our study points to aspects of clinical care that could be considered in clinical decision-making because of their impact on QOL and not necessarily on survival outcomes.

Glioblastoma (GBM) is a type of high-grade glioma (HGG) that accounts for half of the primary brain and central nervous system (CNS) cancer diagnoses and is highly malignant and difficult to treat.^1^ The overall survival (OS) is less than 2 years despite standard treatment, which includes a multimodal approach of maximum safe resection followed by chemoradiotherapy and adjuvant chemotherapy.^2^

Patients who are older than 65 years or frail have been studied to evaluate de-escalated or condensed treatment courses, such as hypofractionated radiotherapy (RT) or chemotherapy alone.^3–12^ For patients without O^6^-methylguanine-DNA methyltransferase (MGMT) promoter hypermethylation, chemotherapy may not provide benefit, and RT alone may be recommended.^13^ This latter population is often not eligible for therapeutic clinical trial enrollment due to their poor Karnofsky Performance Scale (KPS). Thus, treating clinicians are left with little guidance on how to best manage these patients, seeking to balance disease control and treatment-related toxicity.^14^

Specialty palliative care has consistently been shown to decrease pain and symptom distress while increasing quality of life (QOL) for patients with cancer, while also demonstrating measurable benefits for family caregivers.^15–17^ Despite the high symptom burden and national recommendations for integration of specialty palliative care throughout standard oncology care, as a standalone intervention or in conjunction with cancer-directed treatment, these patients have both low utilization with and late engagement with palliative care and associated resources.^18–21^ Likewise, caregivers are overburdened and experience distress through the patient’s disease course.^22–25^ Early specialty palliative care interventions have been shown to be helpful in other cancer types but have yet to be more deeply integrated into the clinical care of GBM.^26–28^ Patients with functional disability in the setting of GBM are likely to have high symptom burden and may benefit from early specialty palliative care intervention.

In this study, we report our institutional experience during 2013-2023 of treatment modalities and outcomes of patients with newly diagnosed GBM and poor KPS who were receiving chemoradiotherapy in the inpatient setting. We characterize treatment patterns, outcomes, and factors associated with survival and discharge disposition in this understudied population, with the goal of informing future clinical trials and treatment guidelines about the importance of considering patient-centered outcomes as additional factors beyond progression-free and OS.

## Methods

### Ethics and Patients

We conducted a comprehensive retrospective study of adult patients with newly diagnosed GBM and poor KPS who were admitted to the inpatient service and treated at Memorial Sloan Kettering Cancer Center (MSK) between January 2013 and December 2023. The study was performed under waiver by the Institutional Review Board, not requiring patient consent.

Eligibility criteria included patients aged 18 years or older with KPS <70, a diagnosis of GBM, and admitted to MSK, during which they received at least 1 fraction of RT for GBM within 60 days from the date of diagnosis. The diagnosis was determined by histological analysis, and, where available, molecular findings via next-generation sequencing analysis, based on CNS WHO classification at the date of diagnosis and age of the patient at diagnosis (Figure S1).^29^ The date of diagnosis was based on the first positive surgical pathology specimen. Karnofsky Performance Scale was used to measure functional status, using either explicit clinical documentation or carefully estimated values based on a comprehensive review of clinical notes, with inclusion of patients with KPS < 70. KPS was collected whenever it was documented or estimated by careful review of clinical notes and based on neurological exam at the time of admission to Neurology, during which RT was delivered. The response to surgical intervention was captured from the clinical examination and impression. Review of neurologic examination documentation included particular attention to cognitive domains, including level of consciousness, behavior, and language. Patients who received RT while inpatient for recurrent disease were excluded. Patients were also excluded if there was another active malignancy or a known cancer predisposition syndrome.

### Analytic Variables

Demographic variables were collected, including age, sex, race, and preferred language. Radiation therapy details were collected, including planned and delivered treatment mode, dose, and fractionation schedule. Information on systemic chemotherapies used during hospitalization was collected. Advanced care planning (ACP) documentation, including resuscitation status and healthcare proxy information, and the presence of specialty palliative care consultation were also captured. Specialty palliative care consults were included if they occurred prior to discharge. The ability of each patient to participate in healthcare decision-making was determined an by in-depth review of ACP and other clinical notes.

Vital status and dates of death or last available follow-up were determined. Survival measures were calculated based on the date of diagnosis and the last RT session until the date of death or censor.

### Statistical Analysis

Descriptive statistics such as medians, ranges, and proportions were used to characterize the cohort. Overall survival was estimated using the Kaplan-Meier method. For primary analyses, since all patients lived to receive radiation, follow-up time was calculated from the date of the last radiation treatment until death for those with events or last follow-up for those who were censored. OS from the date of diagnosis was also explored. Univariable Cox regression models were performed to investigate the associations between variables of interest with OS. The association of bevacizumab with OS was performed using a time-dependent variable in the Cox regression model. Univariable logistic regression models were performed to investigate the associations between variables of interest with discharge to home. Multivariable models were built with variables that were statistically significant in the univariable setting. All tests were two-sided with a level of statistical significance set at <0.05. Statistical analyses were performed using SAS v9.4 (The SAS Institute, Cary, NC) and R v4.4.2 (The R Foundation for Statistical Computing).

## Results

### Patient Population

**Figure 1.**
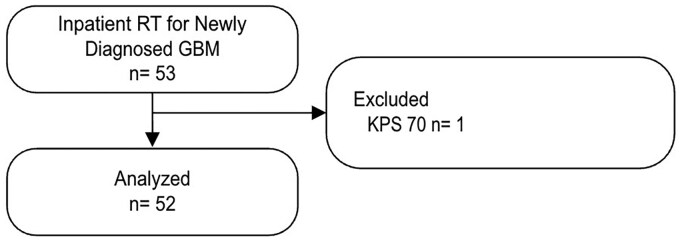
CONSORT diagram. Consolidated Standards of Reporting Trials (CONSORT) diagram of the retrospective study design. The institutional database was queried between January 2013 and December 2023 for multiple concurrent parameters including newly diagnosed glioblastoma (GBM), any fraction of radiotherapy (RT) while hospitalized, and age ≥18 at the time of diagnosis. Patients were then excluded if RT was for the purpose of recurrent disease or more than 60 days from initial diagnosis. One patient, who was excluded for Karnofsky Performance Scale (KPS) of 70, received inpatient treatment due to social situation.

We assembled a retrospective case series consisting of 52 eligible patients, who were included in the present study (Figure 1). Median age was 68 years (range: 21-85), with 15/52 (29%) 75 years or older, and 27 (52%) were women (Table 1). Median KPS was 50 (range: 30-60); in 10/52 (19%) patients, KPS was estimated by careful review of clinical notes and neurological exam. Prior to the start of RT 39/52 (75%), patients had an abnormal level of consciousness, behavior, or language. Patients identified as 40/52 (77%) non-Hispanic white, 5/52 (10%) Black or African American, 2/52 (4%) Asian American and Pacific Islander, and 5/52 (10%) declined to answer. Pathological and imaging assessment revealed that 27/52 patients (52%) had multifocal discontiguous disease, and 35/52 (67%) had multiple brain regions affected (Figure S1 and Table S1). Regarding care locations, 27/52 (52%) patients had surgery performed outside institutions and were transferred to MSK. In the cohort, 29/52 (56%) underwent biopsy, 22/52 (42%) underwent subtotal resection, and 1/52 (2%) had gross total resection. In the post-operative period, there were 37/52 (71%) clinically unchanged, 8/52 (15%) displaying a decline, and 7/52 (13%) who improved. Those with improvement post-operatively were later admitted or unable to be discharged due to insufficient improvement for discharge (3/7), intratumoral hemorrhage (1/7), traumatic cerebral hemorrhage (1/7), bowel perforation (1/7), and progressive disease (1/7). There were no patients with functional improvement among those who underwent biopsy without resection. Clinical decline post-operatively was attributable to status epilepticus (3/8), delirium (2/8), intratumor hemorrhage (1/8), respiratory failure (1/8), and bacteremia (1/8).

**Table 1.** Patient and tumor characteristics

Characteristic	*n* = 52
Age, years, median (range)	68 (21, 85)
Female, *n* (%)	27 (52%)
Race, *n* (%)	
White	40 (77%)
Black or African American	5 (10%)
Asian and Pacific Islands	2 (4%)
Declined to answer	5 (10%)
KPS, *n* (%)	
Median	50
60	14 (27%)
50	18 (35%)
40	15 (29%)
30	5 (10%)
Mental status change, *n* (%)	39 (75%)
Altered level of consciousness or behavior	34
Aphasia	5
Extent of surgery, *n* (%)	
Biopsy	29 (56%)
Subtotal resection	22 (42%)
Gross total resection	1 (2%)
Diagnosis, *n* (%)	
Glioblastoma	50 (96%)
High-grade glioma	2 (4%)
IDH status, *n* (%)	
Wild-type	46 (88%)
Unknown	6 (12%)
MGMT promoter status, *n* (%)	
Hypermethylated	15 (29%)
Unmethylated	22 (42%)
Unknown	15 (29%)

IDH, isocitrate dehydrogenase; MGMT, O^6^-methylguanine-DNA methyltransferase.

Histological diagnosis was GBM in 50/52 (96%), with 2/52 (4%) as other HGG. Of the patients tested by immunohistochemistry or next-generation sequencing analysis, 46/46 (100%) were negative for isocitrate dehydrogenase (IDH1) R132H mutation or IDH1/2 mutations, respectively (Figure S1). O^6^-methylguanine-DNA methyltransferase promoter hypermethylation was tested in 37/52 and present in 15/37 (41%).

### Treatment, Length of Stay, and Outcomes

**Figure 2.**
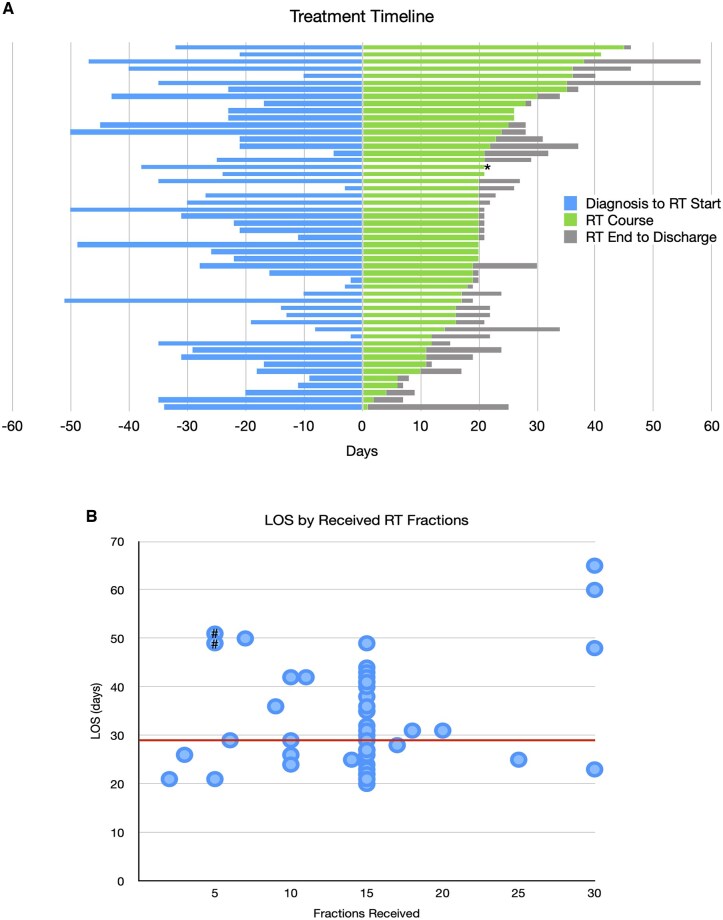
Length of stay vs fractions received. (A) Treatment timeline for all patients. Stacked bar chart demonstrating key timepoints for all patients with days between diagnosis and starting radiotherapy (RT), until completion of RT, and until discharge. For the 1 patient who completed RT outpatient, the last timepoint was set as zero days (marked with *). (B) Shown is Length of Stay (LOS), in days, versus RT fractions received. Median LOS of 29 days marked with red line. Patients who have more fractions than LOS were due to the RT course starting in the outpatient setting and converting to inpatient due to clinical decline. For the 2 patients with long LOS and 5 or less fractions (marked with #), the LOS were both due to delay in diagnosis.

All 52 patients received RT (Table 2, Table S2). The median number of days from diagnosis to RT start was 23 days (range: 2-51) (Figure 2A). There were 8 patients who started RT in the outpatient setting, then had clinical decline, and completed the remaining treatment inpatient. For these patients 1 was planned for full fractionation and the other 7 were for hypofractionated courses; the median time between RT start and admission was 9 days (range: 1-23). For the overall cohort, the median planned dose was 40.05 Gy over 15 fractions. Forty-six patients (88%) completed the planned RT course. Concurrent temozolomide (TMZ) was administered to 50 (96%) patients and bevacizumab to 12 (23%) patients; all patients who received bevacizumab also received TMZ.

**Table 2.** Treatments received

Characteristic	*n* = 52
Days from diagnosis to RT start, median (range)	23 (2, 51)
Radiation plan (Gy, fractions), *n* (%)	
60, 30	6 (12%)
40.05, 15	31 (60%)
Other plans (Table S2)	15 (29%)
Dose received, Gy, median (range)	40.05 (7, 60)
Number of fractions received, median (range)	15 (2, 30)
Adjunctive treatments, *n* (%)	
Temozolomide	50 (96%)
Bevacizumab	12 (23%)

RT, radiotherapy.

At the time treatment began, decisions were made by the patient alone in 5/52 of cases (10%), the patient and surrogate in 16/52 (31%), or a surrogate decision-maker without the patient’s participation in 31/52 (60%) (Table 3). For those 31 cases where the patient was not able to contribute to the decision-making process, in 9/31 cases (29%), decisions were made by a previously appointed Healthcare Agent (HCA), and 22/31 (71%) decisions were made by a next of kin. Patients who were unable to participate in decision-making commonly had impairments in cognition, including 27/31 (87%) with abnormal levels of consciousness or behavior and 4/31 (13%) with aphasia in the absence of alteration in consciousness or behavior. Sixteen patients (31%), or their decision maker(s), elected against cardiopulmonary resuscitation efforts as part of ACP prior to discharge. Specialty palliative care consultation was provided to 16/52 (31%) patients: 8 occurred prior to the start of RT. The 16 patients were non-overlapping. Palliative care domains addressed were complex goals of care 16/16 (100%), symptom management 11/16 (60%), patients and caregiver psychosocial support 8/16 (50%), and end-of-life care 1/16 (6%). The median time between diagnosis and palliative care consultation was 25 days (range: 4-81) with 1 patient having consultation prior to diagnosis.

**Table 3.** Decision-maker and palliative care

Characteristic	*n* = 52
Who was the decision maker?	*N* (%)
Patient	5 (10%)
Patient with surrogate	16 (31%)
Surrogate without patient	31 (60%)
GOC discussion by palliative care specialist	16 (31%)
Advance directive at discharge	
Yes	16 (31%)
No	36 (69%)

GOC, Goal(s) of care.

The median length of stay (mLOS) was 29 days (range: 20-65) (Figure 2B). The median time between the last fraction of RT and discharge was 3 days (range: 0-24), with 1 patient discharged during the treatment course and completed the remainder as an outpatient.

Following treatment, 11/52 (21%) patients were discharged home, 28/52 (54%) to acute or subacute rehabilitation facilities, 8/52 (15%) to inpatient hospice, 4/52 (8%) to home hospice, and 1/52 (2%) patient died during hospitalization.

**Figure 3.**
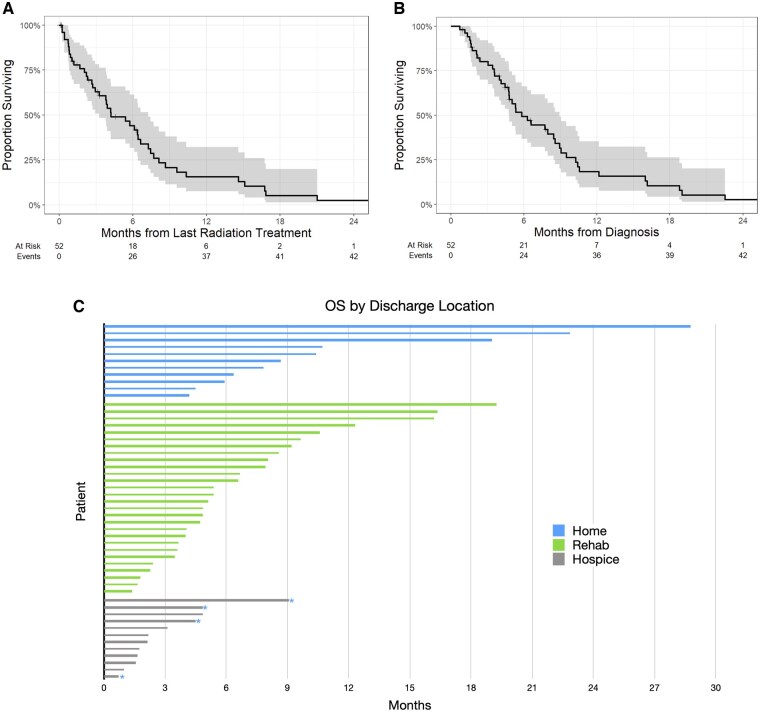
Overall survival and discharge location. (A) Kaplan-Meier curve demonstrating overall survival (OS) of the study population as measured from the last RT session. (B) Kaplan-Meier curve demonstrating OS of the study population as measured from diagnosis. (C) OS of the study population sorted by discharge location. Patients that returned home under Hospice at Home are represented in the Hospice group (marked with blue *).

Median follow-up for survivors from the last radiation treatment was 2.4 months (range: 0.07-6.25). The median OS (mOS) from last RT was 4.21 months (95% CI, 2.93-6.63) and from diagnosis was 5.82 months (95% CI, 4.75-8.55) (Figure 3). The proportion surviving was 44.2% (95% CI, 29.4%-57.9%) at 6 months and 15.6% (95% CI, 6.5%-28.4%) at 12 months following last RT. There were statistically significant univariable associations between OS and higher KPS and higher received dose of RT, but when adjusted for KPS, the effect from RT dose received was no longer significant (Table 3A). There was no effect seen on survival from age, extent of resection, or use of bevacizumab. Patients who were younger and had higher KPS had associations with higher likelihood of discharge to home, but when adjusted for KPS, the effect of age was no longer significant (Table 3B). Overall, KPS was the sole factor that associated with survival and ability to be discharged home.

## Discussion

Our study describes patients with newly diagnosed GBM who received cancer-directed treatment while hospitalized due to functional disability and low KPS. In this group, most patients never left the hospital between their initial surgery and the start of chemoradiation. The few patients who were able to return home after surgery had worsening symptoms shortly after surgery and were readmitted within a median of 9 days. There was a high completion rate of planned treatment, though few patients were able to return home at the time of discharge. While there have been previous descriptions of prognostic factors on OS for patients with GBM, such as age, performance status, extent of surgery, and molecular features such as MGMT promoter methylation status, in our study, KPS at admission remained the sole predictor of ability to return home and survival.^30^ Patients with newly diagnosed GBM and low KPS have an urgent unmet need for specialty palliative care involvement. Further, providers face a dilemma guiding those who are unable to return home after surgery, as placement in rehabilitation facilities may lead to significant delays in the initiation of cancer-directed therapies since these treatments are often not able to be delivered while these patients are at rehab.

There is little guidance on how to best care for patients with poor performance status, particularly for those who are unfit for outpatient cancer-direct treatment. Additionally, some studies combine old and frail patients versus poor performance alone.^7^ ^,^^9^^,^^12^ Our study provides important insights into the clinical characteristics, treatment approaches, and poor outcomes of patients with newly diagnosed GBM and low KPS unable to receive outpatient cancer-directed treatment. Our cohort displays features typical of the broader IDH wild-type GBM population and included a significant proportion of patients with features that traditionally correlate with more aggressive disease progression, including higher rates of biopsy-only cases and unmethylated MGMT promoter status. All patients received cancer-directed therapy, including RT, and the vast majority also received TMZ.

This cohort is enriched in intensity of cancer-directed treatment by virtue of the selection for patients receiving RT inpatient, though also likely due to other selection factors. Patients and their caregivers advocated for all treatment avenues to be considered, as demonstrated by the self-initiated transfer of more than half to our center. While not directly captured, we can draw inferences about the emotional and existential drives to seek out cancer-directed treatment largely by the patient’s caregivers that correspond with previous findings.^27^ This desire for additional treatment further amplifies the knowledge gaps about how to best care for patients and their caregivers in this context and clinical decision-making about which patients would benefit from aggressive cancer-directed treatment. While a minority in this group, there were 6 patients who lived longer than 15 months from diagnosis. There were no clear factors that would portend a better prognosis in this subgroup, with a similar median age, KPS, extent of resection, disease involvement, MGMTp status, RT received, and discharge location.

The diagnosis of GBM has a dramatic impact not only on patients but also on their caregivers, who suddenly must make complex healthcare decisions. Indeed, all patients in our cohort had low KPS, and most were cognitively impaired. Thus, most of the clinical decisions did not involve patients, given their severely limited decision-making capacity associated with disease- and treatment-associated neurological decline. In our study, more treatment decisions were made without patient participation (60%) compared to decisions made by the patient, either alone or with a surrogate (40%).

Integrating specialty palliative care in conjunction with cancer-directed therapy is recommended per oncology guidelines and supports care delivery that is aligned with patients’ and families’ goals and health-related values.^21^ In the outpatient setting, a multi-front approach with collaboration between the patient’s primary oncologist and palliative care specialist, as described by Desai et al.^1^ can normalize and systematize early palliative integration into cancer care. Such strategies may be additionally beneficial for those seen in this cohort, where there is early identification of a worse prognosis. Our results aim to raise awareness to all members of the clinical team caring for these patients and inform clinical practice in the future. Taken together, a comprehensive and multidisciplinary treatment plan, including specialty palliative care involvement, should be incorporated no later than the initial identification of functional disability in the context of this disease and recommended to all patients. Such an approach would ensure upstream attempts to capture the patient’s goals, values, and care preferences to assist with care planning if future decision-making capacity becomes compromised. Specialty palliative care is also likely to complement other forms of psychological and social support for caregivers as they navigate complex goals of care decisions, particularly for those unable to elicit direct input from the patient.^32^

When considering discharge patterns, most were not able to return home and instead remained in a skilled nursing setting. Given the intrinsic limitations of this retrospective study, we could not determine the ability of these patients to return home prior to death. Future studies should carefully assess and annotate these outcomes. Post-discharge palliative care interventions have been studied for other advanced solid tumor cancers to help patients and caregivers in the home setting to reduce end-of-life hospital readmissions, which may be applied to those with GBM.^33–35^

Previous studies have described an association between delays in starting treatment and poor outcomes.^36–38^ To help mitigate this effect, our study excluded patients if the date of admission was greater than 2 months from the date of surgery. The median duration from diagnosis to treatment start (23 days) was within the clinical timeline. The short time between RT completion and discharge (median of 3 days) likely indicates there were no other barriers to discharge than RT itself. The effect of the short mOS seen in our cohort is compounded by patients spending a considerable amount of their remaining survival time in the index hospitalization. The 6- and 12-month survivorship was low, although it is not known who is more likely to benefit from these treatments that may contribute to disease stability or extend survival. There has been optimization of 5 or fewer fraction regimens, which could be considered in this population to decrease length of stay (LOS) and overall treatment burden.^10^^,^^39^^,^^40^ If appropriate, further RT for select patients could be considered as subsequent courses.^41^^,^^42^ A chemotherapy-only plan may be appropriate for patients with MGMT promoter hypermethylation, though our cohort was enriched for unmethylated status, where chemotherapy is predicted to provide little or no benefit, and RT remains the only cancer-directed treatment with demonstrated benefit.^13^ Additionally, often rehabilitation facilities will not permit chemotherapy during the stay, and patients may still face treatment delays. There is likely variability in treating these patients, and further inquiry of multidisciplinary practice patterns more broadly may provide a better understanding.

Some limitations of the present study include the relatively small sample size, variation in RT plan, and heterogeneity in the use of bevacizumab. We also were not able to find comparative cohorts with such detailed clinical annotation and follow-up to validate our findings in external cohorts, which speaks of the uniqueness of our dataset but at the same time suggests that controlled studies should be conducted in a prospective manner. Although the rate of palliative care consultation in our study was low, it is institutional practice for the neuro-oncology team to address goals of care in line with treatment decisions prior to consulting specialty palliative care. These discussions may not have been consistently captured in our retrospective review due to the variability in documentation. Our analysis does not offer any data on QOL after discharge, even among the small group of patients who survive beyond 12 months. These constraints suggest the need for future research exploring more comprehensive molecular characterization, abbreviated therapeutic approaches, patient-reported outcomes, and refined predictive models for patients with poor performance status.

Finally, our study contributes to the understanding of GBM management in functionally disabled patients. It also underscores the limitations of considering OS and PFS as the main factors determining decision-making in practice, with complex multidisciplinary decisions to be made. We highlight the need for the prospective evaluation of patient-centered outcomes, with the interest of condensing or de-escalating treatment even if the survival benefit is not substantial but may lead to shortened LOS and improved QOL. These findings might help refine trial criteria and design for these patients, where survival outcomes should be accompanied by a comprehensive evaluation of improvements in patient-centered outcomes.

## Supplementary Material

npaf109_Supplementary_Data

## Data Availability

Due to patient privacy restrictions, patient data and protected health information from this study are not publicly available. All other data will be made available upon reasonable request from a qualified medical or scientific professional for the specific purpose laid out in that request and may include deidentified individual and/or pooled participant data. The data for this request will be available after a data access agreement has been signed.
