# Supplementary material for: Outcomes of inpatient chemoradiotherapy for patients with newly diagnosed glioblastoma and significant functional impairment
Source: Neurooncol Pract. 2025 Oct 24;13(2):312–21. doi: 10.1093/nop/npaf109 (PMC13153683; doi:10.1093/nop/npaf109)
Supplement: npaf109_Supplementary_Data [file npaf109_supplementary_data.zip › Sielski_Supplemental 091725 - FINAL.pdf]

SUPPLEMENTAL FILE

Title: Outcomes of Inpatient Chemoradiotherapy for Patients with Newly Diagnosed Glioblastoma and Significant Functional Impairment

Supplementary Tables

1. Tumor Location and Affected Regions

2. Additional RT Plans

3. Univariable Analyses

Supplementary Figure Legend

1. Representative Neuroimaging and Pathologic Findings

SUPPLEMENTAL TABLES

Supplementary Table 1. Tumor Location and Affected Regions. Listed are categorization of the extent of disease. Multifocal disease was determined by review of pre-operative reports and imaging. Descriptions of the brain structures or regions that were involved by disease or significant vasogenic edema.

Extent of disease	No. (%)
Unifocal	25 (48%)
Multifocal	27 (52%)
Brain structures involved	
Left hemisphere	30 (58%)
Right hemisphere	37 (71%)
Bihemispheric	18 (35%)
Supratentorial midline and deep nuclei	25 (48%)
Brainstem	8 (15%)
Cerebellum	5 (10%)
Cerebral lobes involved	
Frontal	33 (63%)
Temporal	24 (46%)
Parietal	16 (31%)

Occipital	11 (21%)

Supplemental Table 2. Additional RT Plans. Listed are RT plans outside 60 Gy in 30 fractions or 40.05 Gy in 15 fractions, sorted by planned fractions.

Record ID	Planned cGy	Planned Fractions
**25**	5000	25
**9**	4300	18
**16**	3600	18
**11**	3571	15
**1**	4400	14
**10**	3000	10
**24**	3500	10
**34**	3500	10
**47**	3000	10
**4**	1400	7
**27**	1602	6
**33**	3000	5
**48**	3000	5
**49**	3500	5
**36**	1400	4

Supplemental Table 3. Univariable Analyses. A) Univariable Cox Regression from Last Radiation Treatment. Age, sex, resection, MGMT hypermethylation, LOS, and Bevacizumab were not statistically significantly associated with overall survival from last radiation treatment. Higher KPS, higher received Gy, and more received fractions each were statistically significantly associated with a decreased risk of death.

B). Univariable predictors of Home as Discharge Location. Sex, Resection, received RT Gy and fractions, receipt of a palliative/supportive care consultation, MGMT status, receipt of inpatient Avastin, and patient involvement in decision-making were not associated with discharging to home. Older patients were statistically significantly less likely to discharge to home (OR=0.94, 95%CI: 0.88-0.99). Higher KPS patients were statistically significantly more likely to discharge to home (OR=1.11, 95%CI: 1.03-1.21).

Supplemental Table 3A

**Characteristic**	**HR**	**95% CI**	**p-value**
Age at admission, years	1.023	0.999-1.048	0.066
Gender: Female	Reference	Reference	Reference
Gender: Male	1.13	0.61-2.08	0.70
Resection: Biopsy only	Reference	Reference	Reference
Resection: STR/GTR	1.02	0.55-1.88	0.96
KPS at Admission	0.95	0.91-0.98	0.003
Dosed Gy	0.9996	0.9993-0.9999	0.01
Received Fractions	0.93	0.87-0.99	0.03

MGMT Hypermethylation: No	Reference	Reference	Reference
MGMT Hypermethylation: Yes	0.72	0.34-1.55	0.40
LOS, days	1.02	0.99-1.05	0.15
Bevacizumab: No	Reference	Reference	Reference
Bevacizumab: Yes (time-dependent)	0.79	0.38-1.64	0.52

Abbreviations: HR: hazard ratio, CI: confidence interval, STR: subtotal resection, GTR: gross total resection, KPS: Karnofsky performance scale, MGMT: O6-methylguanine-DNA methyltransferase promoter, LOS: length of stay.

Supplemental Table 3B

**Characteristic**	**OR**	**95% CI**	**p-value**
Age at admission, years	0.94	0.88-0.99	0.04
Gender: Female	Reference	Reference	Reference
Gender: Male	0.92	0.27-3.09	0.90
Resection: Biopsy only	Reference	Reference	Reference
Resection: STR/GTR	0.53	0.14-1.79	0.32
KPS at Admission	1.11	1.03-1.21	0.01
Dosed Gy	1.00	1.00-1.00	0.24
Received Fractions	1.05	0.95-1.17	0.31
Received palliative/supportive care consultation: No	Reference	Reference	Reference
Received palliative/supportive care consultation: Yes	0.46	0.09-1.78	0.29
MGMT Hypermethylation: No	Reference	Reference	Reference

MGMT Hypermethylation: Yes	0.97	0.21-4.23	0.97
Bevacizumab: No	Reference	Reference	Reference
Bevacizumab: Yes	3.44	0.88-13.8	0.07
Patient involved: No	Reference	Reference	Reference
Patient involved: Yes	0.66	0.18-2.24	0.51

Abbreviations: OR: odds ratio, CI: confidence interval, STR: subtotal resection, GTR: gross total resection, KPS: Karnofsky performance scale, MGMT: O6-methylguanine-DNA methyltransferase promoter.

SUPPLEMENTAL FIGURE

Supplemental Figure 1. Representative Neuroimaging and Pathologic Findings

Demonstrated are representative imaging prior to radiotherapy (RT). Where available contrast enhanced MRI is shown with T1 post-contrast and T2/FLAIR axial images. For patients with multifocal or extensive disease the area of largest disease burden is shown. Pathologic findings are reported including histologic diagnosis, WHO 2021 integrated molecular diagnosis, and relevant markers of Isocitrate dehydrogenase (IDH) and O^6-methylguanine-DNA methyltransferase (MGMT) promoter methylation status. Patients without IDH testing were due to testing done prior to inclusion as standard practice (ID 1, 2, 3, 8, 30), inconclusive immunohistochemistry (IHC) staining by outside facility (ID 46), and insufficient material for molecular confirmation (ID 47).

Supplemental Figure 1. Neuroimaging and Pathologic Findings

Record ID	Pre-Radiotherapy MRI		Histological Diagnosis	Molecular diagnosis by WHO 2021	IDH Status	MGMT Status
	T1 Post-contrast	T2/FLAIR				
**1**	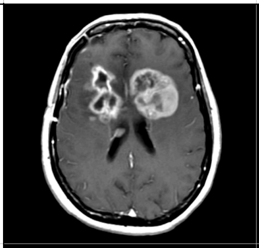	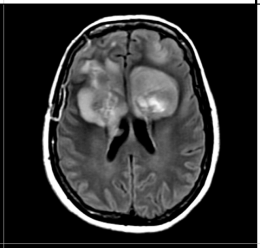	Glioblastoma	High Grade Glioma NOS	Not performed	Not performed
**2**	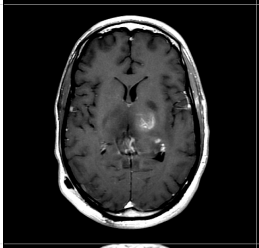	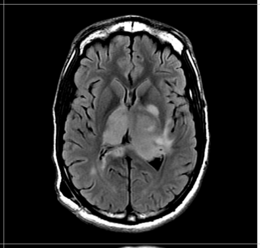	Glioblastoma	High Grade Glioma NOS	Not performed	Unmethylated
**3**	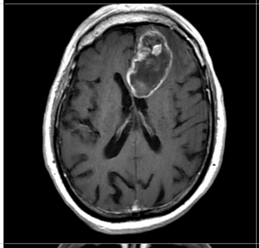	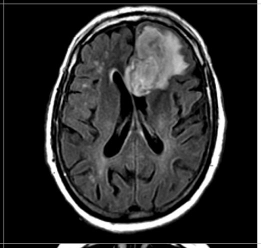	Glioblastoma	High Grade Glioma NOS	Not performed	Not performed
**4**	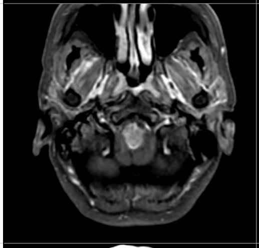	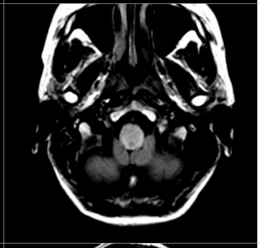	Anaplastic Astrocytoma	Glioblastoma, IDH wild-type	Wild-type, by IHC	Unmethylated
**5**	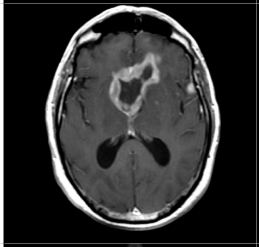	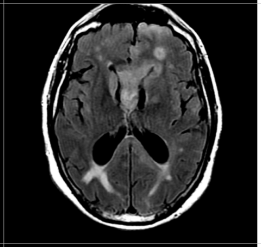	Glioblastoma	Glioblastoma, IDH wild-type	Wild-type, by IHC	Not performed
**6**	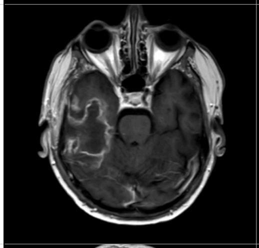	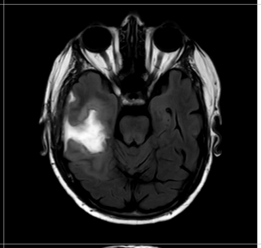	Glioblastoma	Glioblastoma, IDH wild-type	Wild-type, by ICH & NGS	Not performed
**7**	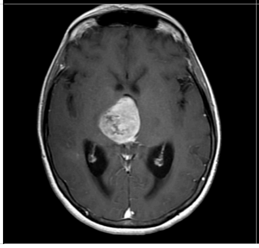	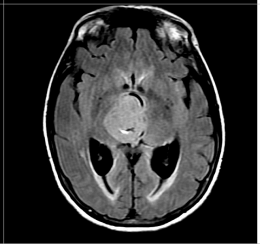	Glioblastoma	Glioblastoma, IDH wild-type	Wild-type, by IHC	Not performed

Record ID	Pre-Radiotherapy MRI		Histological Diagnosis	Molecular diagnosis by WHO 2021	IDH Status	MGMT Status
	T1 Post-contrast	T2/FLAIR				
**8**	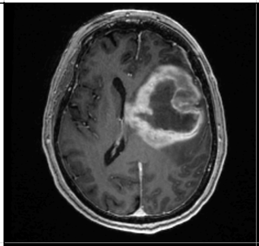	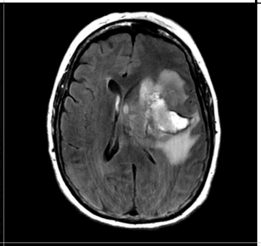	Glioblastoma	High Grade Glioma NOS	Not performed	Unmethylated
**9**	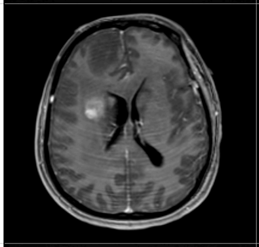	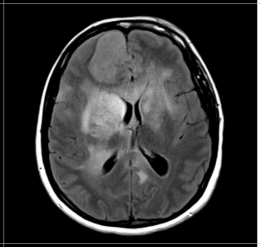	Anaplastic Astrocytoma	Glioblastoma, IDH wild-type	Wild-type, by IHC	Unmethylated
**10**	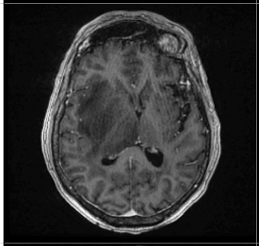	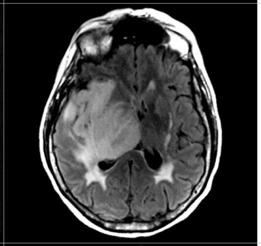	Anaplastic Astrocytoma	Glioblastoma, IDH wild-type	Wild-type, by IHC	Unmethylated
**11**	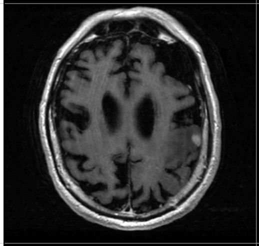	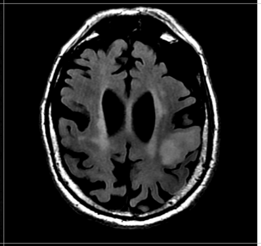	Glioblastoma	Glioblastoma, IDH wild-type	Wild-type, by ICH & NGS	Hypermethylated
**12**	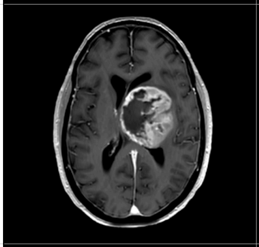	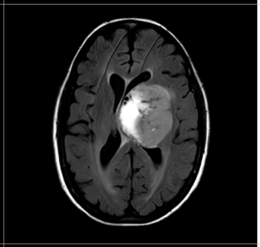	Glioblastoma	Glioblastoma, IDH wild-type	Wild-type, by IHC	Not performed
**13**	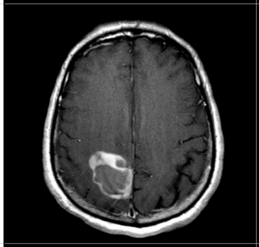	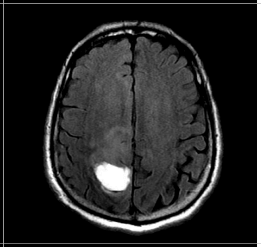	Glioblastoma	Glioblastoma, IDH wild-type	Wild-type, by IHC	Not performed
**14**	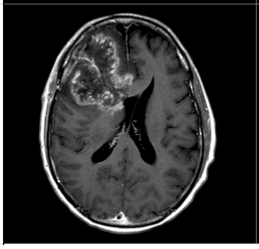	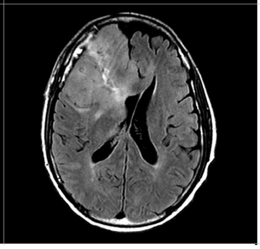	Glioblastoma	Glioblastoma, IDH wild-type	Wild-type, by IHC	Unmethylated

Record ID	Pre-Radiotherapy MRI		Histological Diagnosis	Molecular diagnosis by WHO 2021	IDH Status	MGMT Status
	T1 Post-contrast	T2/FLAIR				
**15**	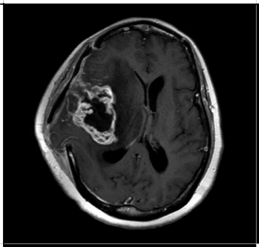	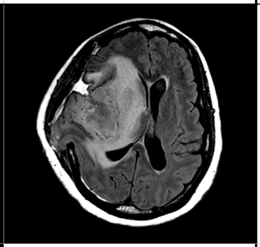	Glioblastoma	Glioblastoma, IDH wild-type	Wild-type, by IHC	Unmethylated
**16**	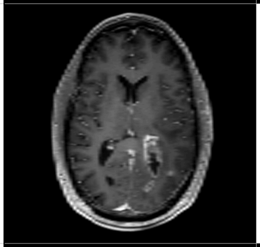	X	Glioblastoma	Glioblastoma, IDH wild-type	Wild-type, by ICH & NGS	Unmethylated
**17**	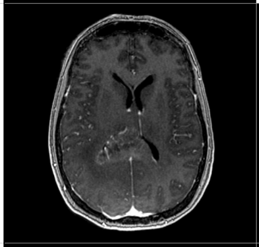	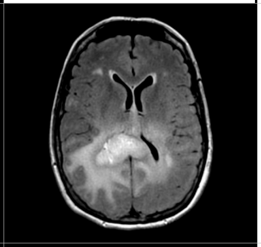	Glioblastoma	Glioblastoma, IDH wild-type	Wild-type, by ICH & NGS	Unmethylated
**18**	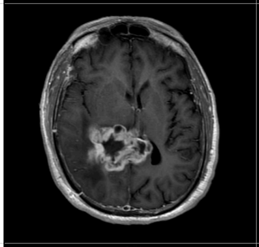	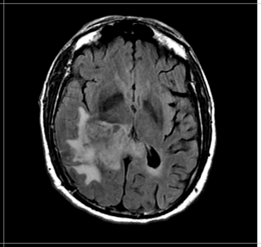	Glioblastoma	Glioblastoma, IDH wild-type	Wild-type, by IHC	Unmethylated
**19**	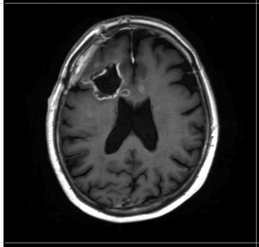	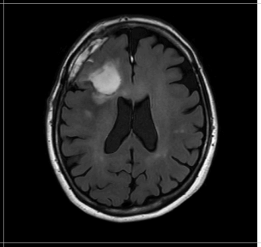	Glioblastoma	Glioblastoma, IDH wild-type	Wild-type, by ICH & NGS	Hypermethylated
**20**	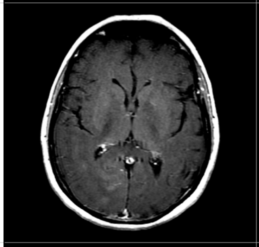	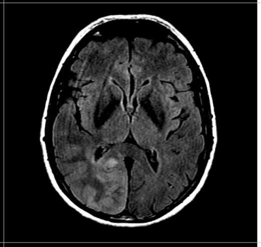	Glioblastoma	Glioblastoma, IDH wild-type	Wild-type, by ICH & NGS	Unmethylated
**21**	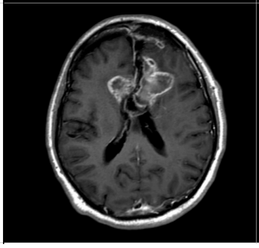	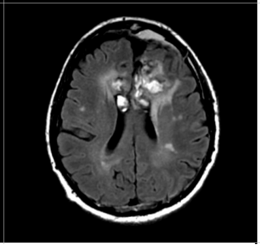	Glioblastoma	Glioblastoma, IDH wild-type	Wild-type, by ICH & NGS	Unmethylated

Record ID	Pre-Radiotherapy MRI		Histological Diagnosis	Molecular diagnosis by WHO 2021	IDH Status	MGMT Status
	T1 Post-contrast	T2/FLAIR				
**22**	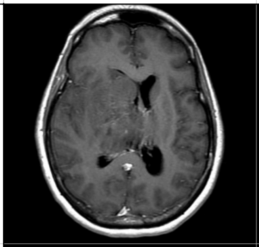	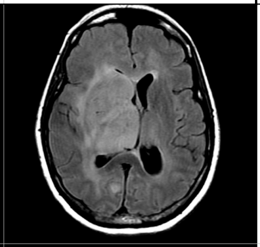	Anaplastic Astrocytoma	Glioblastoma, IDH wild-type	Wild-type, by ICH & NGS	Not performed
**23**	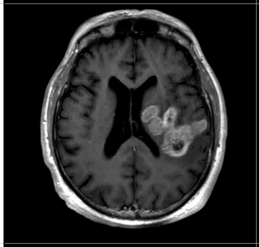	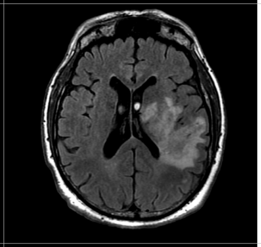	Glioblastoma	Glioblastoma, IDH wild-type	Wild-type, by IHC	Not performed
**24**	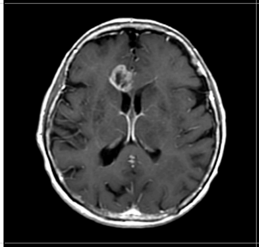	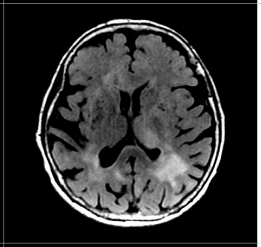	Glioblastoma	Glioblastoma, IDH wild-type	Wild-type, by ICH & NGS	Not performed
**25**	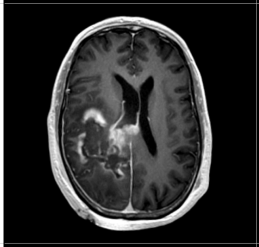	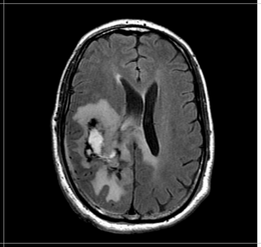	Glioblastoma	Glioblastoma, IDH wild-type	Wild-type, by ICH & NGS	Hypermethylated
**26**	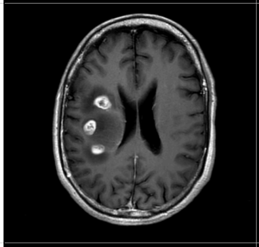	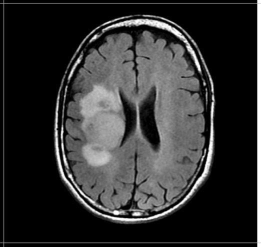	Glioblastoma	Glioblastoma, IDH wild-type	Wild-type, by ICH & NGS	Unmethylated
**27**	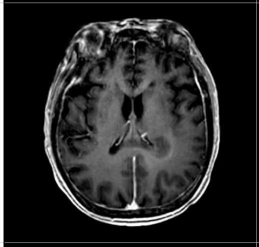	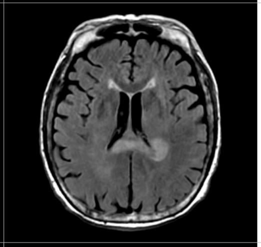	Glioblastoma	Glioblastoma, IDH wild-type	Wild-type, by IHC	Not performed
**28**	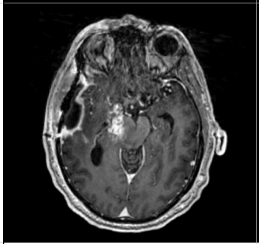	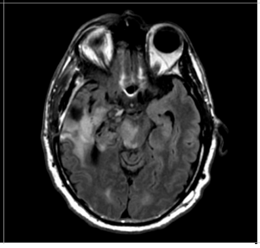	Gliosarcoma	Glioblastoma, IDH wild-type	Wild-type, by ICH & NGS	Unmethylated

Record ID	Pre-Radiotherapy MRI		Histological Diagnosis	Molecular diagnosis by WHO 2021	IDH Status	MGMT Status
	T1 Post-contrast	T2/FLAIR				
**29**	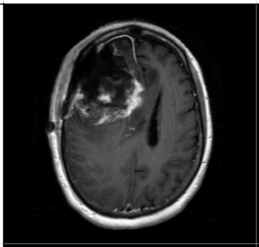	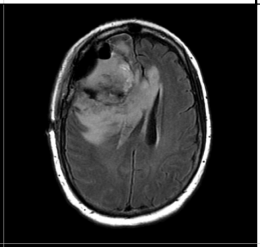	Glioblastoma	Glioblastoma, IDH wild-type	Wild-type, by ICH & NGS	Unmethylated
**30**	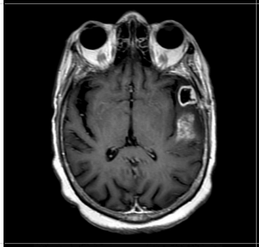	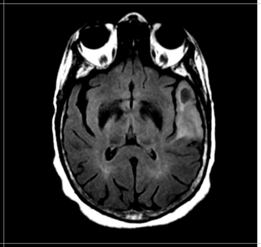	Anaplastic Astrocytoma	High Grade Glioma NOS	Not performed	Not performed
**31**	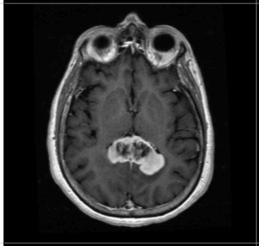	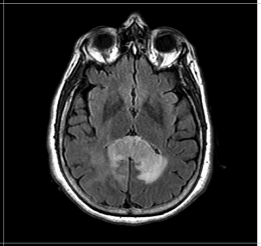	Glioblastoma	Glioblastoma, IDH wild-type	Wild-type, by ICH & NGS	Unmethylated
**32**	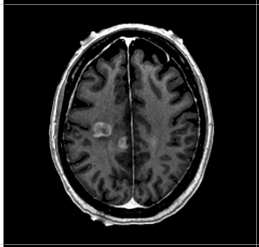	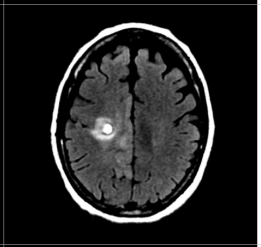	Glioblastoma	Glioblastoma, IDH wild-type	Wild-type, by ICH & NGS	Hypermethylated
**33**	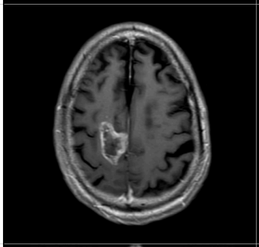	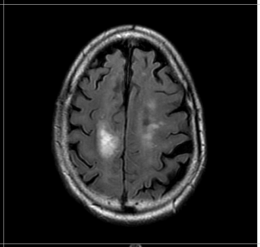	Glioblastoma	Glioblastoma, IDH wild-type	Wild-type, by ICH & NGS	Unmethylated
**34**	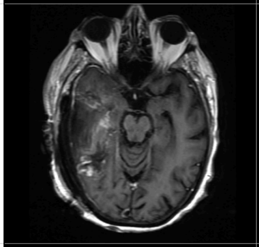	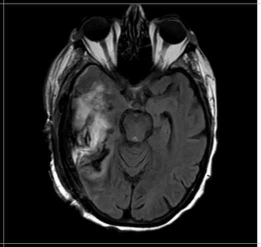	Glioblastoma	Glioblastoma, IDH wild-type	Wild-type, by ICH & NGS	Unmethylated
**35**	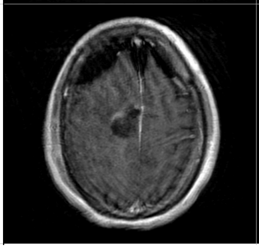	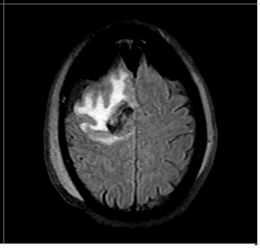	Glioblastoma	Glioblastoma, IDH wild-type	Wild-type, by ICH & NGS	Hypermethylated

Record ID	Pre-Radiotherapy MRI		Histological Diagnosis	Molecular diagnosis by WHO 2021	IDH Status	MGMT Status
	T1 Post-contrast	T2/FLAIR				
**36**	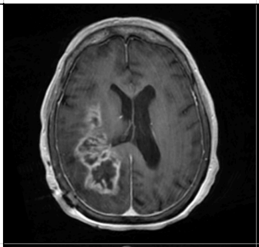	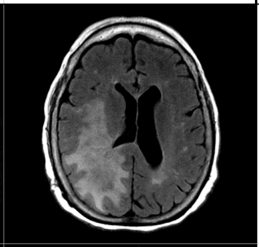	Glioblastoma	Glioblastoma, IDH wild-type	Wild-type, by IHC	Unmethylated
**37**	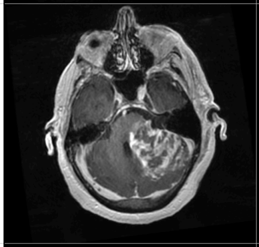	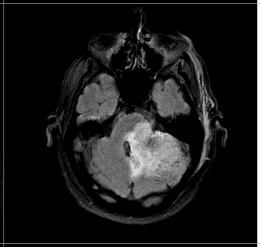	Glioblastoma	Glioblastoma, IDH wild-type	Wild-type, by IHC	Hypermethylated
**38**	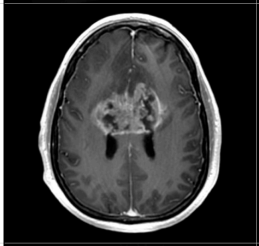	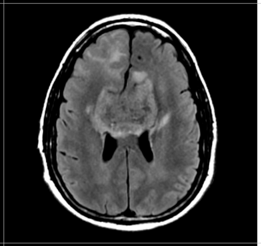	Glioblastoma	Glioblastoma, IDH wild-type	Wild-type, by IHC	Not performed
**39**	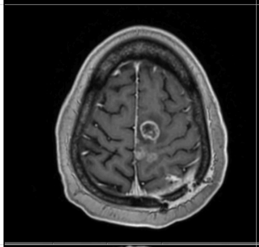	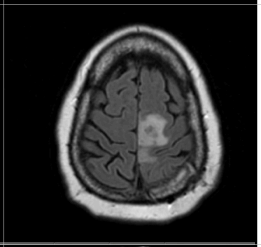	Glioblastoma	Glioblastoma, IDH wild-type	Wild-type, by ICH & NGS	Hypermethylated
**40**	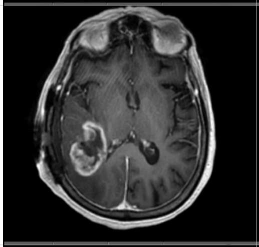	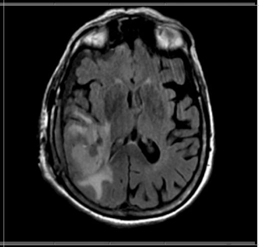	Glioblastoma	Glioblastoma, IDH wild-type	Wild-type, by IHC	Hypermethylated
**41**	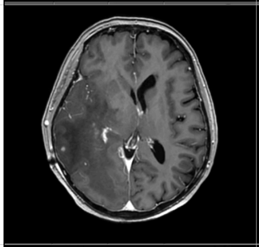	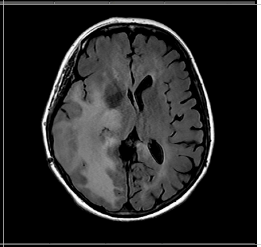	Glioblastoma	Glioblastoma, IDH wild-type	Wild-type, by ICH & NGS	Hypermethylated
**42**	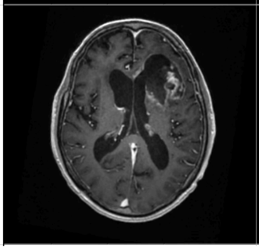	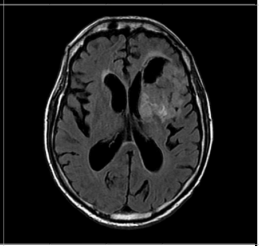	Glioblastoma	Glioblastoma, IDH wild-type	Wild-type, by ICH & NGS	Unmethylated

Record ID	Pre-Radiotherapy MRI		Histological Diagnosis	Molecular diagnosis by WHO 2021	IDH Status	MGMT Status
	T1 Post-contrast	T2/FLAIR				
**43**	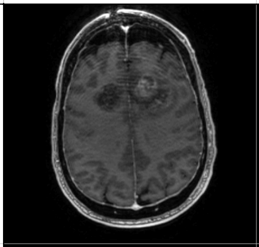	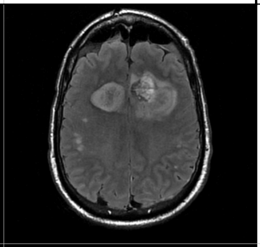	Glioblastoma	Glioblastoma, IDH wild-type	Wild-type, by ICH & NGS	Hypermethylated
**44**	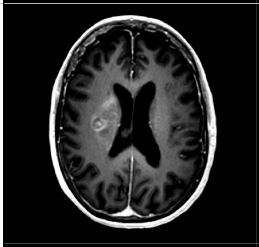	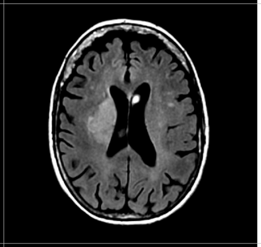	Glioblastoma	Glioblastoma, IDH wild-type	Wild-type, by ICH & NGS	Hypermethylated
**45**	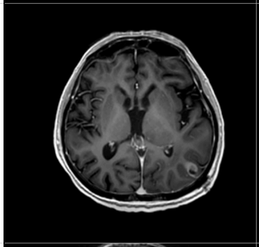	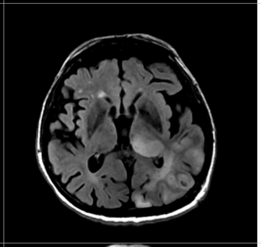	Glioblastoma	Glioblastoma, IDH wild-type	Wild-type, by ICH & NGS	Hypermethylated
**46**	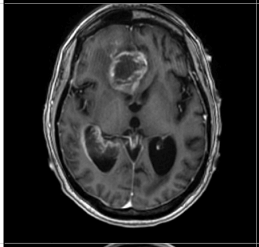	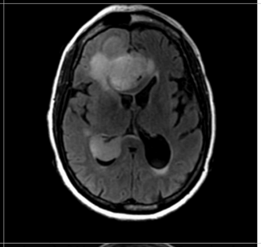	Glioblastoma	High Grade Glioma NOS	Not performed	Not performed
**47**	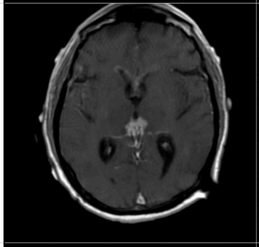	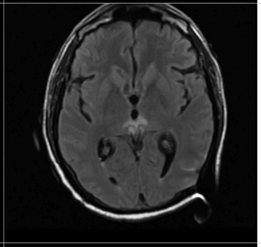	High Grade Glioma, IDH wild-type, H3 wild-type	High Grade Glioma NEC	Wild-type, by IHC & NGS	Not performed
**48**	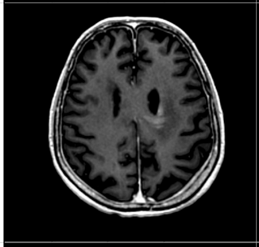	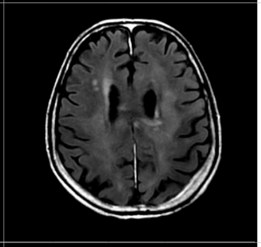	Glioblastoma	Glioblastoma, IDH wild-type	Wild-type, by ICH & NGS	Unmethylated
**49**	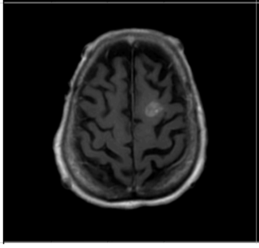	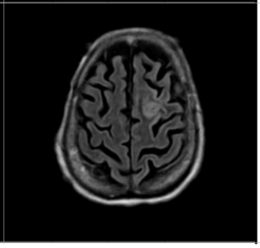	Glioblastoma	Glioblastoma, IDH wild-type	Wild-type, by ICH & NGS	Unmethylated

Record ID	Pre-Radiotherapy MRI		Histological Diagnosis	Molecular diagnosis by WHO 2021	IDH Status	MGMT Status
	T1 Post-contrast	T2/FLAIR				
**50**	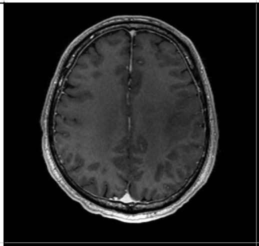	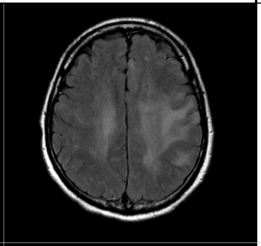	Glioblastoma	Glioblastoma, IDH wild-type	Wild-type, by ICH & NGS	Hypermethylated
**51**	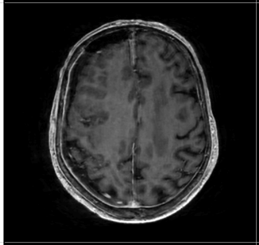	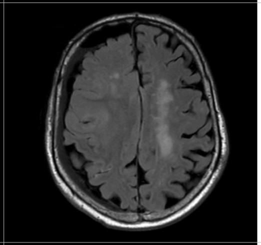	Glioblastoma	Glioblastoma, IDH wild-type	Wild-type, by ICH & NGS	Hypermethylated
**52**	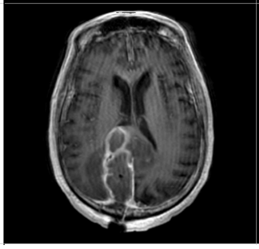	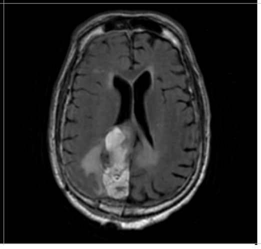	Glioblastoma	Glioblastoma, IDH wild-type	Wild-type, by ICH & NGS	Hypermethylated

IDH *Isocitrate dehydrogenase*
MGMT *O^6-methylguanine-DNA methyltransferase*
NOS *Not otherwise specified*
NEC *Not elsewhere classified*
IHC *Immunohistochemistry*
NGS *Next-generation sequencing*
